# Trend in health-related physical fitness for Chinese male first-year college students: 2013–2019

**DOI:** 10.3389/fpubh.2023.984511

**Published:** 2023-03-01

**Authors:** Xiaoxi Dong, Fan Huang, Gerene Starratt, Zheyi Yang

**Affiliations:** ^1^Department of Physical Education, Chongqing University of Posts and Telecommunications, Chongqing, China; ^2^Faculty of Education, University of Macau, Macau, China; ^3^Adrian Dominican School of Education, Barry University, Miami, FL, United States; ^4^Physical Education Institute, Shijiazhuang University, Shijiazhuang, China

**Keywords:** physical fitness, a natural experiment design, multivariate analysis of variance, public health, college students

## Abstract

**Introduction:**

Physical fitness is a health indicator contributing to the prevention of non-communicable diseases that threaten public health. Studies across a number of global populations indicate that physical fitness is generally declining. This study investigated the trend in physical fitness of Chinese male first-year college students from 2013 to 2019 to offer critical information for fostering individual and public health.

**Methods:**

This study used archival data and a natural experiment design capturing 4 years of data prior to implementation of the Healthy China 2030 initiative and 3 years following. Physical fitness tests were based on the Chinese national student physical fitness standards for males including body mass index, vital capacity, standing-long-jump, sit-and-reach, pull-ups, 50 m sprint, and 1,000 m run. Because the physical fitness tests set different standards for males and females, female data will be reported separately. Data from a total of 3,185 Chinese male first-year college students from a private university in Hebei Province of China were included in the study. A one-way multivariate analysis of variance was used for analyzing the research data.

**Results:**

The results indicated an overall significant difference in health-related physical fitness of Chinese male first-year college students, with scores on health indicators generally declining from 2013 to 2019. Despite improvement on some fitness variables in some years, performance on virtually all indicators was diminished compared to baseline years.

**Discussion:**

These findings can contribute to the existing global literature in the field of public health showing general declines in physical fitness. Chinese universities have the opportunity to support Healthy China 2030 goals and cultivate individuals' physical fitness by offering physical education course that encourage college students to participate in moderate-to-vigorous-intensity physical activities in order to support physical fitness development.

## 1. Introduction

Non-communicable diseases (NCDs) are implicated in approximately 41 million deaths each year worldwide, making them a crucial health issue threatening both public and individual health (1). Physical inactivity has been identified both as a risk factor (2) and a cause (3) contributing to fatal NCDs around the globe. Unfortunately, a high level of global prevalence of physical inactivity has been reported (4–7). For instance, in 2018, WHO estimated that more than 20% of adults and 80% of adolescents across the world did not engage in the level of physical activities that WHO (2) guidelines recommend for health. Not surprisingly, the global high prevalence of physical inactivity has imposed a substantial cost burden on the public healthcare system (8). As such, the individual challenge of physical inactivity has also become a public health challenge (9). It is reasonable to assume that strategies that could contribute to increases in physical activity may have the potential to reduce the prevalence of NCDs.

In contrast, physical fitness is a remarkable healthy indicator for preventing critical NCDs, e.g., cardiovascular mortality (10) and vascular structure (11). For example, Clausen et al. (12), who examined midlife cardiorespiratory fitness and all-cause and cardiovascular mortality, found that the level of midlife cardiorespiratory fitness was positively correlated to an increase in longevity and was a significant indicator of protection from all-cause mortality. Moreover, physical fitness is also positively associated with well-being (13), physical activity (14), and academic performance (15). Physical fitness can be conceptualized as a series of an individuals' capabilities of performing and/or participating in physical activity (16).

Physical fitness has been theorized as two types of fitness: skill-related physical fitness and health-related physical fitness (HRPF) (17, 18). Skill-related physical fitness highlights the development of motor skills associated with sports performance, e.g., speed, power, coordination, and agility (17). Arguably, skill-related physical fitness may not be a reliable and appropriate measure providing meaningful information to foster a healthy living for people in general. On the other hand, HRPF emphasizes the essential development of overall health and disease prevention (19). HRPF consists of five components, e.g., body composition, cardiovascular endurance, flexibility, muscular strength, and muscular endurance (16, 20). The effectiveness of HRPF in contributing to overall health and disease prevention for encouraging healthy living has been extensively studied. Findings indicate that the factors of HRPF promote healthy living (10–12). Therefore, HRPF is a critical health factor that can be used to assist with planning for countering the challenge of NCDs while cultivating public health.

College life is a unique period that represents the first major transition from adolescence to early adulthood in life (21), and habitual behavior established in the period would affect people's lifestyles in the future (22). In addition, a decrease in cardiorespiratory fitness measured by a 6-min run test was found among 11–14 years old Croatian children and adolescents from years 1999 to 2014 (23). This decreased cardiorespiratory fitness may cause individuals to be at-risk for unhealthy living in the future. For instance, children with a lower level of cardiorespiratory fitness are likely to experience increased health challenges in obesity and insulin resistance in adulthood (24). One of the possible strategies to overcome the health challenges is physical activity. However, there is a serious concern about engagement in physical activity during college life, due to a lack of participation in physical activity during the transition from adolescence to college life (25). Approximately, four in ten college students inadequately participated in physical activity for promoting health across 23 countries (26). Consequently, this high prevalence of physical inactivity might have a negative effect on HRPF among college students, even with different generations. Kaj et al. (27), who conducted a study to compare Hungarian college students' fitness between 1997–1998 and 2011–2012, found that fitness indicators, such as sit-and-reach, body fat percentages, and sit-up, performed better in the year of 1997–1998 than 2011–2012. The downward trend in HRPF fitness is also found among Chinese medical students from the years 2014 to 2016 (28). Ultimately, this downward trend in HRPF would harm individuals and public health.

In order to cultivate both individual and public health, the guidance of Healthy China 2030 was released by the Ministry of Human Resources and Social Security of the People's Republic of China (29). One of the healthy targets recommended by Healthy China 2030 for improving the health level is to increase the level of HRPF among individuals. However, limited studies have been conducted to examine the differences in HRPF among Chinese first-year college students, especially in relation to the implementation of the Healthy China 2030 guidance designed to improve physical fitness. To address this knowledge gap, the purpose of this study was to investigate the differences in HRPF of Chinese male first-year college students before and after Healthy China 2030 was issued in order to assess the physical fitness indicators as measures of individual and public health. Specifically, the study used a natural experiment design to examine the trends in physical fitness indicators from a baseline average of 4 years before the implementation of Healthy China 2030 (2013–2016) compared to 3 years following (2017–2019). The research question is “Are there differences in HRPF for Chinese male first-year college students between the years 2013 to 2019?” Specifically, it was hypothesized that there would be statistically significant differences in the linear combination of body mass index (BMI), vital capacity, 50 m sprint, standing-long-jump, sit-and-reach, 1,000 m run, and pull-ups for Chinese male first-year college students between the years 2013 to 2019.

## 2. Materials and methods

### 2.1. Participants

The population for the present study was Chinese male first-year college students enrolled in any year from 2013 to 2019. A convenience sampling strategy was used for recruiting universities in Hebei Province of China to provide archival data for this study. In the end, only one private university agreed to participate. The archival data that were obtained included physical fitness records for both male and female Chinese first-year college students from years 2013 to 2019 who were enrolled in variety of departments (e.g., finance, engineering) and physical education, which is a compulsory class for every student in the university. These years were selected in order to have four baseline years of data before the implementation of Healthy China 2030 and 3 years after. This timeframe was also selected to end prior to the start of the COVID−19 pandemic to preserve the integrity of the comparisons. Because female and male college students took different physical fitness tests (e.g., female sit-and-up vs. male pull-ups) during those years, data for female students will be reported separately. The sample in the present study included 3,185 Chinese male first-year college students (age, *M* = 18.00, *SD* = 1.21; BMI, *M* = 21.06, *SD* = 2.86) who were enrolled in the participating university between 2013 and 2019. The number of participants and descriptive statistics for dependent variables (DVs) are shown in Table 1. The unequal sample sizes for each year occurred naturally as a result of the variation in college enrollment for the university every year. According to Tabachnick and Fidell (30), the unequal sample sizes could not pose specific issues with one-way analysis. Hence, no specific concern was raised with the minor unequal sample sizes for each year.

**Table 1:** Descriptive statistics for dependent variables by year (*N* = 3,185).

	**BMI**	**Vital**	**50 m**	**SLJ**	**SR**	**1,000 m**	**Pull-ups**
**Year 2013 (*****n** =* **535)**							
*M*	21.16	4,331.89	7.32	232.82	13.83	231.08	15.73
*SD*	2.73	477.48	0.57	15.56	4.59	24.81	3.97
skewness	0.42	−0.14	−0.06	0.14	0.23	0.09	0.59
kurtosis	−0.41	0.36	−0.69	−0.02	−0.18	−0.98	−0.21
**Year 2014 (*****n** =* **284)**							
*M*	20.50	4,327.96	7.46	223.93	12.80	247.26	10.96
*SD*	2.66	583.15	0.63	17.42	5.07	25.00	4.29
skewness	0.70	0.11	0.33	0.39	0.38	0.29	0.45
kurtosis	−0.05	−0.28	0.02	−0.56	−0.38	0.79	0.53
**Year 2015 (*****n** =* **452)**							
*M*	20.93	4,126.45	7.59	220.43	11.27	255.56	7.09
*SD*	2.80	387.91	0.65	16.76	5.27	29.28	4.70
skewness	0.51	−0.05	0.22	0.46	0.53	0.07	0.59
kurtosis	−0.25	0.24	−0.16	−0.27	−0.08	0.32	0.08
**Year 2016 (*****n** =* **562)**							
*M*	21.13	4,214.94	7.54	222.93	11.09	252.60	9.05
*SD*	2.79	498.37	0.68	17.06	5.00	27.59	5.25
skewness	0.55	0.33	0.31	0.51	0.42	0.32	0.41
kurtosis	−0.15	0.33	0.01	−0.19	−0.32	0.58	−0.29
**Year 2017 (*****n** =* **533)**							
*M*	20.94	3,729.61	7.53	222.02	11.02	257.12	8.46
*SD*	2.89	528.69	0.59	16.07	5.80	31.34	4.83
skewness	0.51	0.42	0.23	0.63	0.55	0.26	0.50
kurtosis	−0.36	0.13	−0.15	0.04	−0.50	−0.19	0.19
**Year 2018 (*****n** =* **394)**							
*M*	21.20	3,862.07	7.62	225.81	11.16	252.03	9.15
*SD*	3.06	636.90	0.68	17.03	6.17	32.55	4.97
skewness	0.38	−0.02	0.38	0.62	0.43	0.27	0.28
kurtosis	−0.60	−0.60	−0.30	−0.43	−0.68	−0.11	−0.26
**Year 2019 (*****n** =* **425)**							
*M*	21.36	4,007.85	7.87	225.65	12.03	270.48	8.40
*SD*	3.04	572.38	0.72	20.15	5.38	28.41	4.74
skewness	0.30	0.43	0.22	0.29	0.38	0.13	0.50
kurtosis	−0.66	0.18	−0.91	−0.96	−0.79	−0.20	0.03
**All (*****N** =* **3,185)**							
*M*	21.06	4,079.60	7.56	224.89	11.85	252.00	9.89
*SD*	2.86	566.33	0.67	17.53	5.42	30.73	5.48
skewness	0.48	0.02	0.31	0.40	0.36	0.24	0.33
kurtosis	−0.37	0.01	−0.14	−0.43	−0.48	0.02	−0.28

Vital, vital capacity; 50 m, 50 m sprint; SLJ, standing-long-jump; SR, sit-and-reach; 1,000 m, 1,000 m run. The same hereinafter.

### 2.2. Physical fitness measures

The physical fitness tests utilized in the study were based on the Chinese national student physical fitness standards, which is a reliable and valid physical fitness test used by different researchers (28, 31). In addition, the physical fitness test battery included height and weight, vital capacity, standing-long-jump, sit-and-reach, pull-ups, 50 m sprint, and 1,000 m run. The participants were required to warm up before taking the physical fitness tests. The physical fitness tests were implemented by the physical education professors at their university.

#### 2.2.1. Test of vital capacity

The test of vital capacity was measured with an air spirometer with a dry and sterilized plastic mouthpiece. The participants took a deep breath and exhaled slowly into the mouthpiece until they could no longer exhale. The air spirometer automatically calculated the maximum volume of air (lung capacity per milliliter) and displayed the results after the blowing was completed. The participants took the test twice with an interval of 15 s, and the better of the two scores was recorded.

#### 2.2.2. Test of standing-long-jump

The participants were asked to stand behind a starting line with their feet apart in a natural stance and to jump forward. In addition, the participants were instructed that both feet should jump simultaneously from the standing position, and no additional movements should be made. The horizontal distance was measured from the trailing edge of the starting point to the trailing edge of the nearest landing point. Each student was permitted three jump attempts. The longest jump was recorded in centimeters.

#### 2.2.3. Test of sit-and-reach

In order to examine the flexibility of the participants, the participants were required to take a sit-and-reach test with an equipment of sit-and-reach box and a sitting position and keeping legs straight. The upper body leaned forward with the arms stretching out straight forward simultaneously and the participants' feet were separated between 10 and 15 cm while pushing forward horizontally against the test board. The fingertips of the participants reached out and gradually pushed the test bar forward until the participants could not push further. Each student was permitted two attempts. The furthest distance was recorded in centimeters.

#### 2.2.4. Test of pull-ups

In order to examine the participants' upper body strength, the test of pull-ups was used. The participants were instructed to hold a bar with their hands the same width as their shoulders to form a straight arm suspension. Subsequently, the participants pulled their entire bodyweight upward only with both arms simultaneously while body and arms stayed straight. Additionally, the participants' bodies could not have additional actions, e.g., swing or wiggle. To complete one pull-up, participants were required to pull themselves up until their lower jaws were beyond the upper edge of the bar. The number of pull-ups was recorded.

#### 2.2.5. Test of 50 m sprint

The participants were directed to the starting line of the 50 m straight track and took the standing position. Moreover, the participant was directed to begin in response to the words “ready-and-go” from the instructor. The timekeeper began recording the time upon the instructor's direction to go. The time was recorded as the moment that the participants passed the finish line with their chests. The time recoding was measured in seconds, accurate to one decimal place. The second digit after the decimal point was rounded in terms of the principle of non-zero into 1. Each participant was permitted two attempts and the better performance was recorded.

#### 2.2.6. Test of 1,000 m run

The participants were asked to take a standing position behind the starting line of the 1,000 m run. The test of the 1,000 m run began with the instruction of ready-and-go given by the instructor. Simultaneously, the timekeepers started recording when the instructor directed the participant to begin. Then, the timekeeper finished the recording when the participants passed the finish line. The time recoding was measured in minutes and seconds, accurate to one decimal place. The second digit after the decimal point was rounded in terms of the principle of non-zero into 1. Each participant only had one attempt to take the test.

#### 2.2.7. Test of body mass index (BMI)

BMI was used to measure a degree of overweight and obesity among participants and the formula used for calculating BMI is weight (kg) / height (m)^2^. The criteria for BMI defined by WHO was used, which is scored as equal and higher than 28 (kg/m^2^) defined as obesity, between 24 and 27.9 defined as overweight, between 18.5 and 23.9 defined as normal weight, and smaller than 18.5 defined as low weight (28). Participants' heights and weights were measured by using an electronic weight and height scale. Participants were instructed to take off their shoes and step on the electronic weight and height scale with standing straight. Then, the electronic measure automatically measured individuals' weights and heights with accurate to 0.1 cm.

### 2.3. Procedures

The research setting was a private university in Hebei province of northern China. Researchers contacted the physical education professors at the department of physical education to obtain consent to share their existing physical fitness tests data from 2013 to 2019. The research data were sent to the researchers after the physical education professors agreed to share their research data and cooperate with the researchers in conducting the study.

During each year for which the study data were supplied, the physical education professors scheduled a time for physical fitness tests with their students based on enrollment in their classes. The physical fitness tests were started after an agreement on the time of physical fitness tests from the students was obtained. In addition, every physical fitness test class varied from 20 to 30 students, and each class needed a maximum of 50 min to complete the physical fitness tests. The physical fitness tests were conducted every October from 2013 to 2019. The study was approved by the Institutional Review Board.

### 2.4. Data analysis

The seven DVs for the study were BMI, vital capacity, 50 m sprint, standing-long-jump, sit-and-reach, 1,000 m run, and pull-ups, while the independent variable (IV) was the different years from 2013 to 2019. To examine the differences in HRPF among Chinese male first-year college students, 4 years of data were averaged (20013–2016) for each variable to serve as baseline prior to the issue of Healthy China 2030 in 2016. The baseline for each variable was then compared to the average scores for each variable for the years 2017, 2018, and 2019. First, the R package *psych* (32) was used to produce descriptive statistics and Pearson correlation coefficient for the study variables. Second, the histogram plot, boxplot, Q-Q plot, and scatter plot were conducted to assess the degree to which the assumptions of multivariate normality, multicollinearity, and multivariate outliers, as this study included a large sample size, recommended by Field et al. (33). The visual results indicated that the assumptions were met. Third, the R package *MASS* (34) was used to conduct a one-way multivariate analysis of variance (MANOVA). According to Field et al. (33), one-way MANOVA can be used to examine whether there is a group discrepancy with a combination of multiple dimensions. Hence, a one-way MANOVA was used to simultaneously examine the differences in the linear combination of the seven DVs between the years 2013 to 2019. Fourth, one-way univariate analysis of variance (ANOVA), as follow-up tests to the significant one-way MANOVA, was used to examine the significance of the multivariate effect on each DV separately. Moreover, an alpha of 0.05 was used to determine statistical significance.

## 3. Results

### 3.1. Descriptive statistics

The descriptive statistics to summarize the study variables by year are displayed in Table 1. Moreover, Pearson's correlation analysis showed that virtually all of the correlations among the seven DVs were statistically significant with the exception of the correlation between BMI and standing-long-jump, as displayed in Table 2. The negative correlations between the DVs of 50 m sprint and 1,000 m run and other four DVs (i.e., vital capacity, standing-long-jump, sit-and-reach, and pullups) simply reflect the fact that shorter running times (better performance) were associated with higher scores (better performance) on the other variables. Ultimately, the participants who tended to do well on any test tended to also do well on the other tests. Furthermore, the ranges of associations between the DVs were below an absolute value of 0.70, which satisfied the assumption related to multicollinearity.

**Table 2:** Correlation matrix of the dependent variables (*N* = 3,185).

	**BMI**	**Vital**	**X50 m**	**SLJ**	**SFB**	**X1,000 mm**	**Pullups**
BMI	1						
Vital	0.06^***^	1					
X50 m	0.05^**^	−0.08^***^	1				
SLJ	−0.11^***^	0.12^***^	−0.37^***^	1			
SFB	−0.01	0.05^**^	−0.1^***^	0.17^***^	1		
X1,000 mm	0.1^***^	−0.13^***^	0.3^***^	−0.29^***^	−0.12^***^	1	
Pullups	−0.18^***^	0.11^***^	−0.25^***^	0.34^***^	0.19^***^	−0.36^***^	1

*** *p* < 0.001,

** *p* < 0.01.

### 3.2. MANOVA analysis

A one-way four-level MANOVA was used to examine the differences in a combination of seven DVs across baseline and the years 2017, 2018, and 2019. Specifically, the average baseline was calculated from four years of data prior to and including 2016 when the guidance of Healthy China 2030 was released. The other three levels represented years 2017, 2018, and 2019. Box's M test was used to examine the equality of variance-covariance matrices, and the Box's M test yielded a significant result ($\chi_{\left[84\right]}^{2}$ = 473.95, *p* < 0.01), which revealed that the dependent covariance matrices were not equal across the four levels from the baseline level to 2019. However, the significant result of the Box's M might be due to the large sample size. According to Field et al. (33), the Box's M test could have significant results with a large sample size even if the dependent covariance matrices were equal across the different levels of the IV. Further, Pillai's Trace test was used to examine the significance of the multivariate effects. The results of Pillai's Trace test indicated a statistically significant difference in mean values for the linear combination of the seven DVs with respect to the HRPF among four levels, years from before 2017 to 2019 (Pillai's = 0.25, *F*_[3,3181]_ = 42.09, *p* < 0.0001, partial η^2^ = 0.25). In addition, the results illustrated that 25% of the total variance of the composite seven DVs could be explained by the four timeframes, from the baseline through to 2019, indicating a large effect size (35).

#### 3.2.1. One-way ANOVA for vital capacity

**Figure 1:**
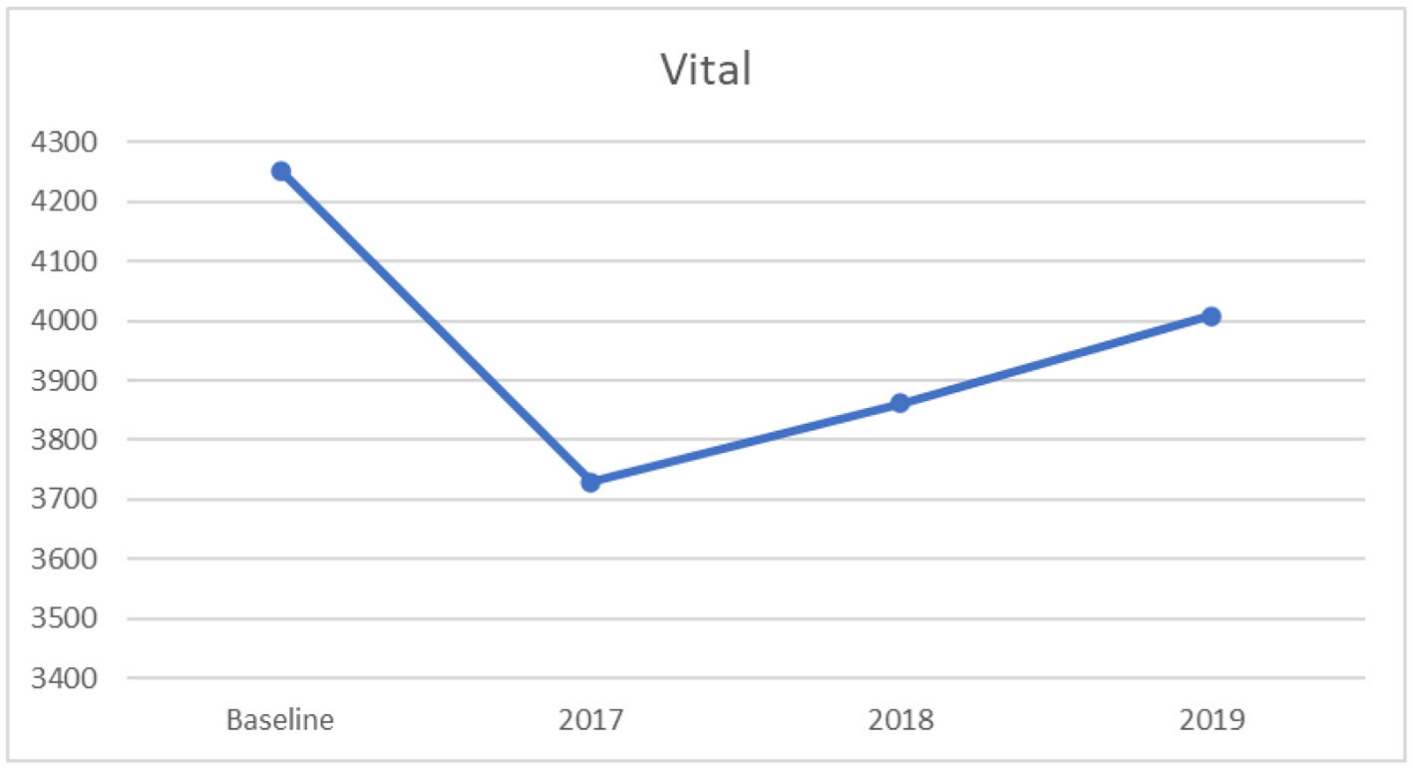
Mean vital capacity scores: across baseline and years 2017–2019.

Subsequently, a series of univariate ANOVAs were conducted to assess the significance of the multivariate effect on each DV among the four timeframes representing the years before 2017 to 2019 separately as follow-up tests to the significant one-way MANOVA. The result indicated a significant univariate effect on vital capacity across the four levels, (*F*_[3,3181]_ = 163.08, *p* < 0.0001, partial η^2^ = 0.13), with a moderate to large effect size, suggested by Cohen (35). Further, Tukey's HSD was conducted to assess the nature of the mean differences in vital capacity between the four levels. The *post-hoc* analysis revealed that the baseline level had significantly higher mean scores in vital capacity than the years 2017 to 2019, as displayed in Table 3. Moreover, the year of 2019 had significantly higher mean scores in vital capacity than the years 2017 and 2018. After a marked drop in the means of the vital capacity from baseline to 2017 was found, an increasing trend from 2017 to 2019 was found, as displayed in Figure 1. For this variable, higher scores indicate better performance.

**Table 3:** Multiple comparisons: mean differences and confidence intervals for vital capacity across mean baseline (2013–2016), 2017, 2018, and 2019.

**Years**	**Mean differences**	***SE***	***t***	**95%CI**	
2017 vs. baseline	−515.16	25.96	−19.85^***^	−581.88	−448.43
2018 vs. baseline	−382.70	29.29	−13.07^***^	−457.99	−307.41
2019 vs. baseline	−236.92	28.40	−8.34^***^	−309.91	−163.92
2018 vs. 2017	132.46	35.05	3.78^***^	42.38	222.54
2019 vs. 2017	278.24	34.30	8.11^***^	190.07	366.41
2019 vs. 2018	145.78	36.89	3.95^***^	50.96	240.60

95% CI, 95% confidence interval. The same hereinafter.

*** *p* < 0.001.

#### 3.2.2. One-way ANOVA for 50 m sprint

**Figure 2:**
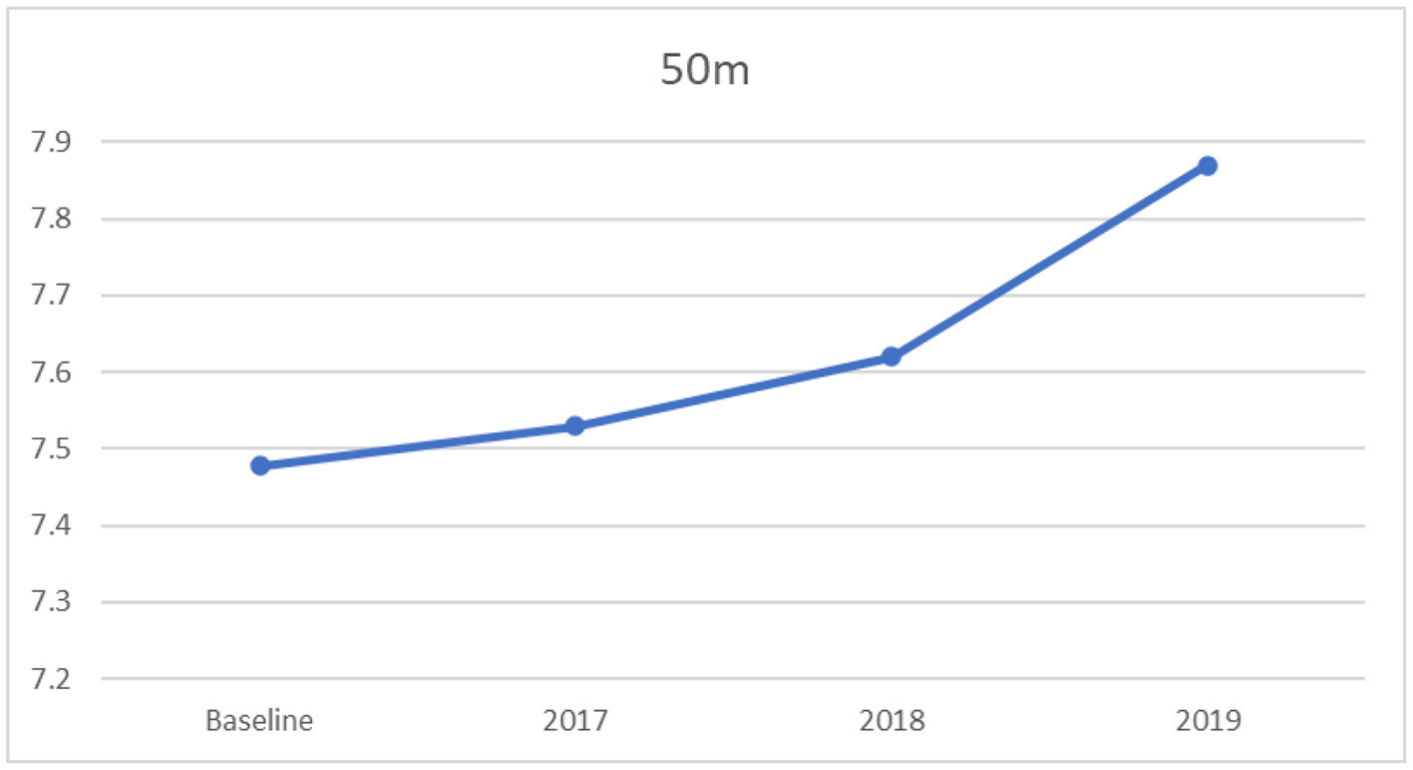
Mean 50 m sprint scores: across baseline and years 2017–2019.

Similarly, there were statistically significant differences in the mean scores of the 50 m sprint across the four timeframes, (*F*_[3,3181]_ = 44.01, *p* < 0.0001, partial η^2^ = 0.04), with a small effect size, according to Cohen (35). The results of the Tukey's HSD revealed that during the baseline years participants took significantly less time on the test of the 50 m sprint than the years 2018 and 2019, as displayed in Table 4. Moreover, participants in the year 2017 took significantly less time on the test of the 50 m sprint than the years 2018 and 2019. There were no significant differences in the mean score on the test of the 50 m sprint between the baseline and 2017. Figure 2 for the 50 m sprint indicated an increasing trend in time on the 50 m sprint test from baseline to 2019. For this variable lower scores indicate better performance.

**Table 4:** Multiple comparisons: mean differences and confidence intervals for 50 m sprint across mean baseline (2013–2016), 2017, 2018, and 2019.

**Years**	**Mean differences**	***SE***	***t***	**95%CI**	
2017 vs. baseline	0.05	0.03	1.57	−0.03	0.13
2018 vs. baseline	0.14	0.04	3.84^***^	0.05	0.23
2019 vs. baseline	0.40	0.04	11.29^***^	0.31	0.49
2018 vs. 2017	0.09	0.04	2.05	−0.02	0.20
2019 vs. 2017	0.35	0.04	8.16^***^	0.24	0.45
2019 vs. 2018	0.26	0.05	5.64^***^	0.14	0.37

*** *p* < 0.001.

#### 3.2.3. One-way ANOVA for standing-long-jump

**Figure 3:**
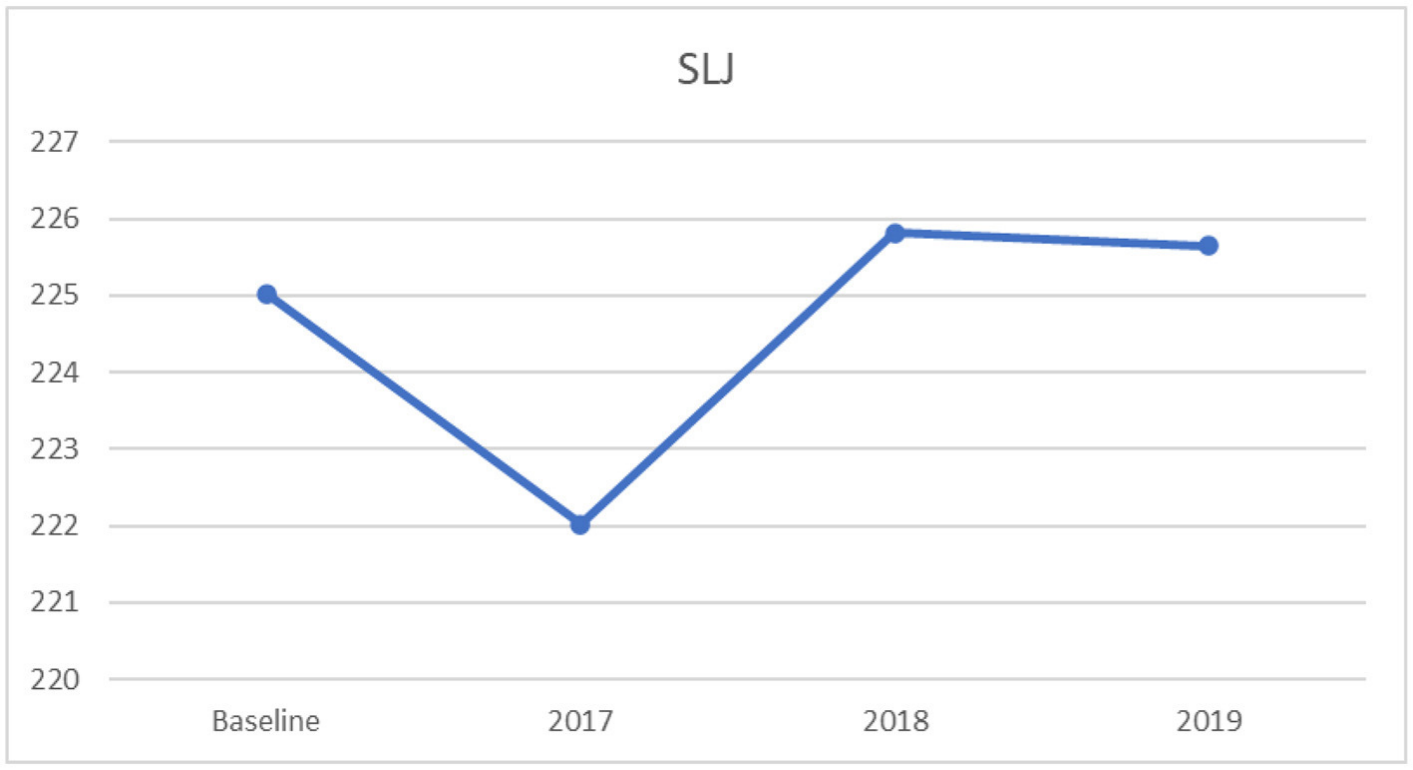
Mean standing-long-jump scores: across baseline and years 2017–2019.

Furthermore, a significant univariate effect was found on standing-long-jump among the four timeframess, (*F*_[3,3181]_ = 5.85, *p* < 0.001, partial η^2^ = 0.005), with less than a small effect size (35). The results of the Tukey's HSD indicated a significantly higher mean score on standing-long-jump at baseline, compare to 2017, as displayed in Table 5. Moreover, the years 2019 and 2018 had significantly higher mean scores in standing-long-jump than the year 2017. There was no significant difference in the mean score on the test of the standing-long-jump across the other years. However, there was an upward-and-downward trend from baseline to 2019, as displayed in Figure 3. For this variable, higher scores indicate better performance.

**Table 5:** Multiple comparisons: mean differences and confidence intervals for standing-long-jump across mean baseline (2013–2016), 2017, 2018, and 2019.

**Years**	**Mean differences**	***SE***	***t***	**95%CI**	
2017 vs. baseline	−3.34	0.86	−3.88^***^	−5.55	−1.12
2018 vs. baseline	0.45	0.97	0.46	−2.05	2.95
2019 vs. baseline	0.30	0.94	0.31	−2.13	2.72
2018 vs. 2017	3.79	1.16	3.26^**^	0.80	6.78
2019 vs. 2017	3.63	1.14	3.19^**^	0.71	6.56
2019 vs. 2018	−0.16	1.22	−0.13	−3.30	2.99

*** *p* < 0.001,

** *p* < 0.01.

#### 3.2.4. One-way ANOVA for sit-and-reach

**Figure 4:**
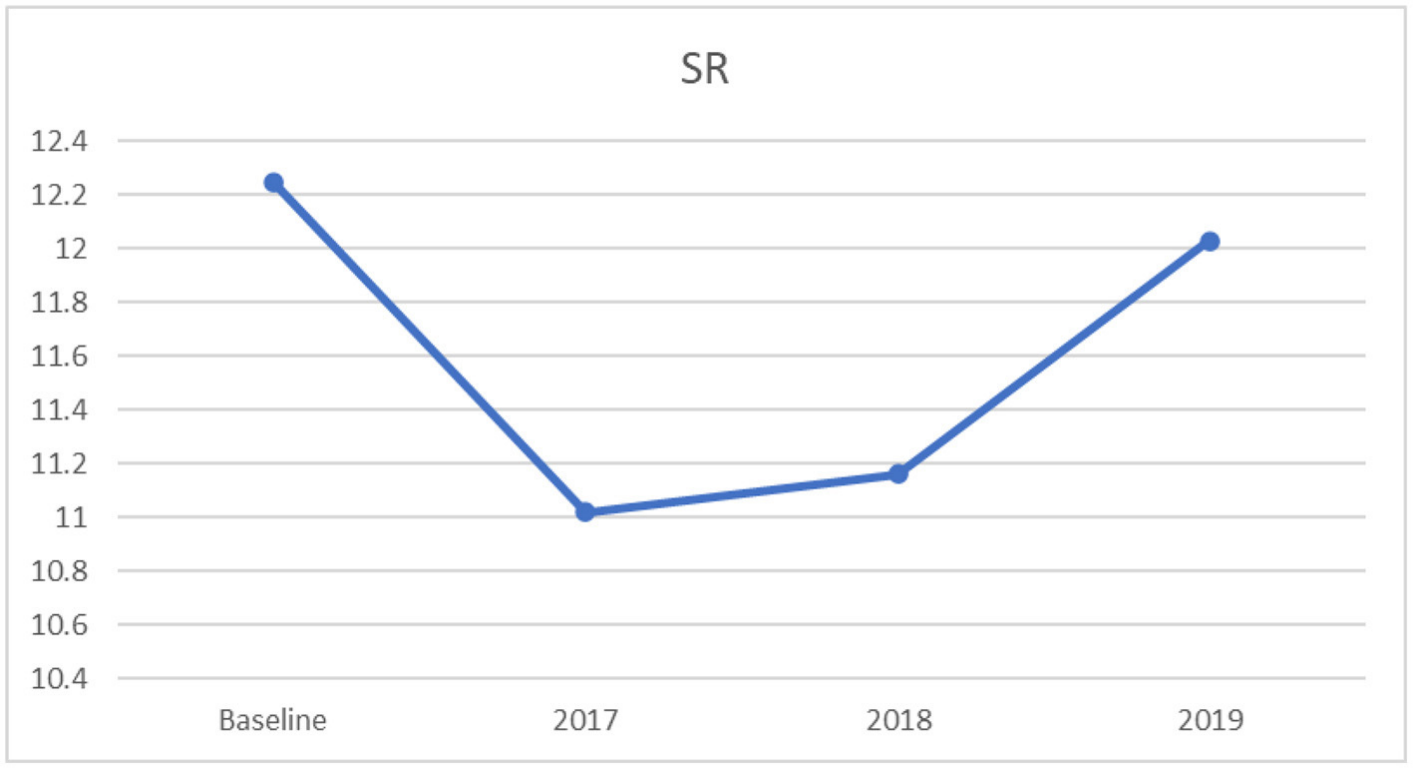
Mean sit-and-reach scores: across baseline and years 2017–2019.

Similarly, a significant univariate effect on sit-and-reach was found across the four timeframes, (*F*_[3,3181]_ = 8.99, *p* < 0.0001, partial η^2^ = 0.008), indicating a less than a small effect size, according to Cohen (35). The results of the Tukey's HSD illustrated that the baseline years had significantly higher mean scores on sit-and-reach than the years from 2017 to 2018, as displayed in Table 6. In addition, the year 2019 had a significantly higher mean score on sit-and-reach compared to 2017. No significant differences were found among other years. Figure 4 shows that sit-and-reach indicated a decreasing curve from baseline to 2017. However, there was a progressing growth from 2018 to 2019. For this variable, higher scores indicate better performance.

**Table 6:** Multiple comparisons: mean differences and confidence intervals for sit-and-reach across mean baseline (2013–2016), 2017, 2018, and 2019.

**Years**	**Mean differences**	***SE***	***t***	**95%CI**	
2017 vs. baseline	−1.17	0.27	−4.42^***^	−1.86	−0.49
2018 vs. baseline	−1.03	0.30	−3.45^**^	−1.81	−0.26
2019 vs. baseline	−0.16	0.29	−0.56	−0.91	0.58
2018 vs. 2017	0.14	0.36	0.39	−0.78	1.06
2019 vs. 2017	1.01	0.35	2.88^*^	0.11	1.91
2019 vs. 2018	0.87	0.38	2.31	−0.10	1.84

*** *p* < 0.001,

** *p* < 0.01,

* *p* < 0.05.

#### 3.2.5. One-way ANOVA for 1,000 m run

**Figure 5:**
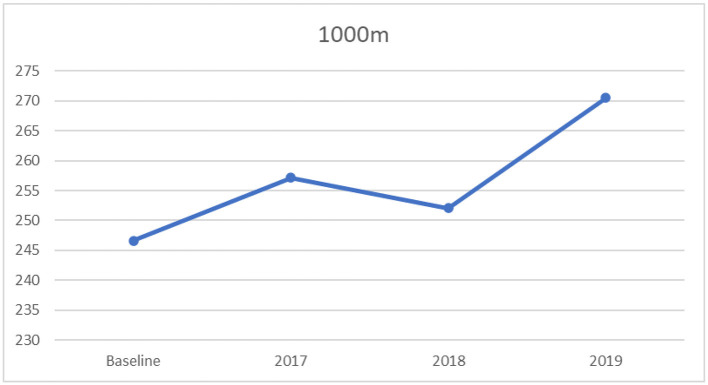
Mean 1,000 m run scores: across baseline and years 2017–2019.

Likewise, the result for the 1,000 m run indicated a significant univariate effect on 1,000 m run among the four timeframes, (*F*_[3,3181]_ = 83.86, *p* < 0.0001, partial η^2^ = 0.073), indicating a moderate to large effect size (35). Further, the results of Tukey's HSD revealed that the baseline had significantly lower mean scores on the test of the 1,000 m run than the years 2017 to 2019, as displayed in Table 7. Additionally, there were significant differences in the mean score of the 1,000 m run between 2017 and 2018, 2017and 2019, and 2018 and 2019. The trend shows increasing time completing the 1,000 m run from before 2017 to 2019, as displayed in Figure 5. For this variable, lower scores indicate better performance.

**Table 7:** Multiple comparisons: mean differences and confidence intervals for 1,000 m run across mean baseline (2013–2016), 2017, 2018, and 2019.

**Years**	**Mean differences**	***SE***	***t***	**95%CI**	
2017 vs. baseline	10.90	1.46	7.48^***^	7.15	14.64
2018 vs. baseline	5.81	1.64	3.54^**^	1.59	10.03
2019 vs. baseline	24.26	1.59	15.23^***^	20.16	28.35
2018 vs. 2017	−5.09	1.97	−2.59^*^	−10.14	−0.03
2019 vs. 2017	13.36	1.92	6.94^***^	8.42	18.31
2019 vs. 2018	18.45	2.07	8.91^***^	13.13	23.77

*** *p* < 0.001,

** *p* < 0.01,

* *p* < 0.05.

#### 3.2.6. One-way ANOVA for pull-ups

**Figure 6:**
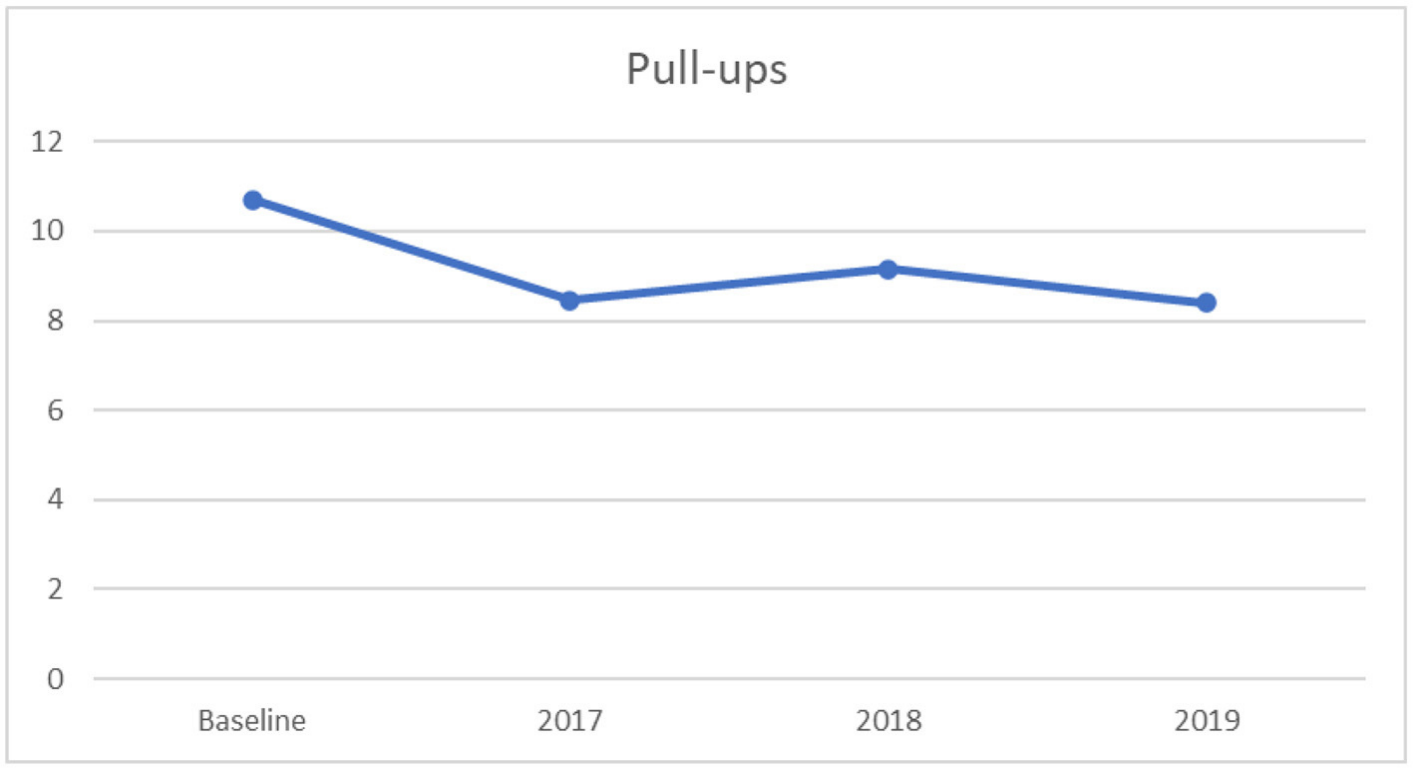
Mean pull-ups scores: across baseline and years 2017–2019.

Moreover, a significant univariate effect on pull-ups was found across the four timeframes, (*F*_[3,3181]_ = 44.08, *p* < 0.0001, partial η^2^ = 0.04), indicating a small effect size, suggested by Cohen (35). The results of Tukey's HSD indicated that the baseline had a significantly higher mean score on pull-ups compared to the years 2017 to 2019, as displayed in Table 8. In addition, there were also significant differences in the mean score of the pull-ups among other years. Further, there was a noticeable declining curve on the mean value of the pull-ups from the year before 2017 to 2019, as displayed in Figure 6. For this variable, higher scores indicate better performance.

**Table 8:** Multiple comparisons: mean differences and confidence intervals for pull-ups across mean baseline (2013–2016), 2017, 2018, and 2019.

**Years**	**Mean differences**	***SE***	***t***	**95%CI**	
2017 vs. baseline	−2.36	0.26	−8.92^***^	−3.04	−1.68
2018 vs. baseline	−1.66	0.30	−5.58^***^	−2.43	−0.90
2019 vs. baseline	−2.41	0.29	−8.34^***^	−3.15	−1.67
2018 vs. 2017	0.69	0.36	1.94	−0.23	1.61
2019 vs. 2017	−0.05	0.35	−0.15	−0.95	0.84
2019 vs. 2018	−0.75	0.38	−1.98	−1.71	0.22

** *p* < 0.001.

#### 3.2.7. One-way ANOVA for BMI

Finally, no significant univariate effect on BMI was found (*F*_[3,3181]_ = 2.57, *p* < 0.053,) among the four levels representing baseline to 2019.

## 4. Discussion

The present study examined the difference in HRPF of Chinese male first-year college students from before and after Healthy China 2030 was issued in 2016 in order to contribute to the conversation about individual and public health. Findings of the present study supported the hypothesis, indicating an overall significant difference in HRPF of the Chinese male first-year college students from baseline to 2019. Despite a fluctuating upward-and-downward trend in HRPF of the study population across some years, overall the findings indicated a general decline in physical fitness The main findings of the present study were consistent with the reports of previous studies conducted by Kaj et al. (27), Chen et al. (28), Pribis et al. (36), and Wetter et al. (37), who found declines in physical fitness among college students. For instance, Pribis et al. (36), reported a decline in physical fitness among college students between 1996 and 2008 in the United States. The factors reported to contribute to the significant decline in HRPF among the student population are varied, e.g., socioeconomic status (38) and eating behavior and patterns (39). According to Wang (40), Chinese college students' lifestyles of leisure-time entertainment have been altered to show an increase in online consumption, e.g., electronic games. Lepp et al. (41) reported that cell phone use was negatively associated with cardiorespiratory fitness among college students. It is reasonable to assume that increased engagement with electronic devices in sedentary activities could be associated with a decline in engagement in physical activities, thus contributing to noted decline in HRPF. Furthermore, Murphy et al. (42) found that Irish university students living away from home were more likely to be categorized in a cluster identified with risky health-related behavior. For the population of Chinese college students, most students have left home to live on campus independently, and one impact of the life-environment transition might be for these students to neglect to maintain and/or cultivate healthy behaviors. Arguably, the living environment of college students would be expected to play a role in fostering HRPF. Hence, special attention to cultivating college students' health-related behaviors during the period of campus living needs to be addressed.

For the purpose of the present study, scores on HRPF indictors were obtained for Chinese male first-year college students for 2013 through 2019. The finding did not reveal a significant difference in body composition as measured by BMI among these years. On the other hand, the findings indicated a decline in lower back flexibility, measured by sit-and-reach, from the years before 2017 to 2019. The present findings are consistent with those studies conducted by Kaj et al. (27), who found a decreasing trend in flexibility among Hungarian college students between 1997–1998 and 2011–2012, and Wetter et al. (37), who reported a decline in flexibility among college students between 2005–2006 and 2010–2011 in the United States. The current findings are consistent with existing research which suggests that these findings may be generalizable beyond this specific population.

It is, however, relevant to note that the present study did show some improvement in some indicators during some timeframes. For example, a gradual increasing trend in lower back flexibility was noted between 2017 and 2019, after a slight decrease trend from years before 2017 to 2017. College students have to take physical education courses as compulsory course, which would have positive influence in college students' lower back flexibility due to first-year students need to do exercise during the college life. Similarly, the present findings showed an increase in vital capacity among Chinese male first-year college students from 2017 to 2019. Moreover, we also revealed improvement in the study population's lower-limb explosive strength, measured by standing-long-jump, among Chinese male first-year college students from 2017 to 2019. While the guidance of Healthy China 2030 was issued in 2016 to encourage educational institutions and public health services to support the development of individuals' physical fitness, mastery of individuals sports skills and engagement in physical activity, the design of this study does not support a causal link between the guidance and these improvements. In fact, despite the improvements in some skills during some timeframes, the overall findings indicate a decline in fitness over the years of the study.

The guidance of Healthy China 2030 consistently highlights the promotion of an individual's physical fitness with various and specific strategies (29). For instance, the guidance encourages cultivating adolescents' physical exercise hobbies, focuses on the mastery of adolescents' in at least one sports skill, and recommends that every student participates in physical activities for at least one hour during school day (29). It would be appropriate for educational institutions and public health services to promote cardiovascular fitness for preventing fatal NCDs, as cardiovascular fitness is associated with a reduction in risk of fatal mortality (43).

The present study did not observe improvement in HRPF, as measured by 50 m sprint, pull-ups and 1,000 m run in current years among the Chinese male first-year college students, despite the growth of vital capacity, flexibility, and standing-long-jump from 2017 to 2019. Specifically, the study population's upper body muscular strength, measured by pull-ups, showed a steep descent from baseline to 2017. Subsequently, the data showed a fluctuating upward-and-downward trend in the compositions of HRPF. Moreover, gradual declines in performance on the 50 m sprint and the 1,000 m run from before 2017 to 2019, as reflected in more time to complete the tests, are consistent with the findings of Chen et al. (28), who found a decreasing trend in physical fitness with a study population of Chinese medical college students from years 2014 to 2016. One possible explanation for the decrease might be related to a high level of prevalence of physical inactivity and sedentary behavior. For example, < 10% of college students engaged in moderate-to-vigorous-intensity physical activity among a population of 4,747 Chinese medical college students (44). Hence, the lack of engagement in moderate-and-vigorous-intensity physical activity might impact the decline in muscular strength and endurance, as moderate-and-vigorous-intensity physical activity is positively associated with physical fitness (45). Although the present study was not able to confirm the association and establish causality, these findings of declines in fitness nonetheless may support a recommendation that educational institutions concentrate on the development of students' muscular strength and endurance in order to sufficiently implement the guidance of Healthy China 2030 as a strategy for coping with the decline in HRPF. The present findings contribute to understanding the trend in HRPF among Chinese male first-year college students.

Although the sample of the present study was from one private college in Hebei Province of China, the study population of students who enrolled at this university had come from different cities in China. Given the alignment of the present finding with previous studies, [e.g., (27, 36, 37)], it is reasonable to assume that these findings would be generalizable at least to similar students enrolled in institutions across China and around the world. To further explore the generalizability of these findings, future research might continue to examine the trend in HRPF with different populations to extend knowledge and inform the development of useful strategies to promote college students' HRPF. To further inform public health policy, future studies might be conducted using an experimental design to examine the cause and effect related to college students' behaviors and HRPF.

The present study reports a trend of a significant decline in HRPF of the Chinese first-year male college students who enrolled in a private university in Hebei Province of China from 2013 to 2019. However, notable findings of the present study reported a gradual increase in the levels of vital capacity, flexibility, and standing-long-jump from 2017 to 2019. The findings describe a manifest downward trend in first-year college male students' muscular strength, muscular endurance, and cardiorespiratory endurance, which might be expected to represent a risk factor that can ultimately contribute to NCDs. Additionally, the declining trend in HRPF among college male students may ultimately represent a negative public health impact as these students age. The theorized causes of the decline in HRPF among college male students are varied, and the possible explanations of the phenomenon may be due to changes in lifestyle, e.g., online consumption (40–42) and a lack of engagement in physical activity (44, 45). In any event, the present findings suggest that individual and public health may benefit from university efforts to continually cultivate individuals' HRPF, especially muscular strength, muscular endurance, and cardiorespiratory endurance. To overcome the declining trend in HRPF, university physical education courses can provide opportunities for college students to engage in moderate-to-vigorous-intensity physical activities in order to fulfill the needs of promoting college students' HRPF. Chinese universities and other educational institutions, with the benefit of guidance provided by Healthy China 2030 might benefit from collaborating with public health services implement programs to cultivate individual engagement in physical activity through exercise hobbies or the mastery of individual sports skills.

## Data availability statement

The raw data supporting the conclusions of this article will be made available by the authors, without undue reservation.

## Ethics statement

The studies involving human participants were reviewed and approved by Barry University. Written informed consent from the participants' legal guardian/next of kin was not required to participate in this study in accordance with the national legislation and the institutional requirements.

## Author contributions

XD, FH, GS, and ZY contributed to conception and design of the study. ZY organized the database. XD and ZY ran the statistics and wrote the first draft of the manuscript. FH and GS revised the manuscript. All authors contributed to manuscript revision, read, and approved the submitted version.
